# Supplementary material for: Predicting visual function by interpreting a neuronal wiring diagram
Source: Nature. 2024 Oct 2;634(8032):113–23. doi: 10.1038/s41586-024-07953-5 (PMC11446822; doi:10.1038/s41586-024-07953-5)
Supplement: Supplementary file 6 — CRF and ERF predictions for individual TmY4 and TmY9 cells. Analogous to Supplementary Data 3, but for TmY target types. Shown are the top four monosynaptic pathways, the strongest pathway passing through each of the top ten intermediary types (ranking from Extended Data Fig. 7), and the trisynaptic pathway Tm1–TmY–Dm3–TmY (see the section entitled Prediction of spatial normalization). [file 41586_2024_7953_MOESM6_ESM.zip › DataS4/TmY9qΓèÑ/720575940613321982.pdf]

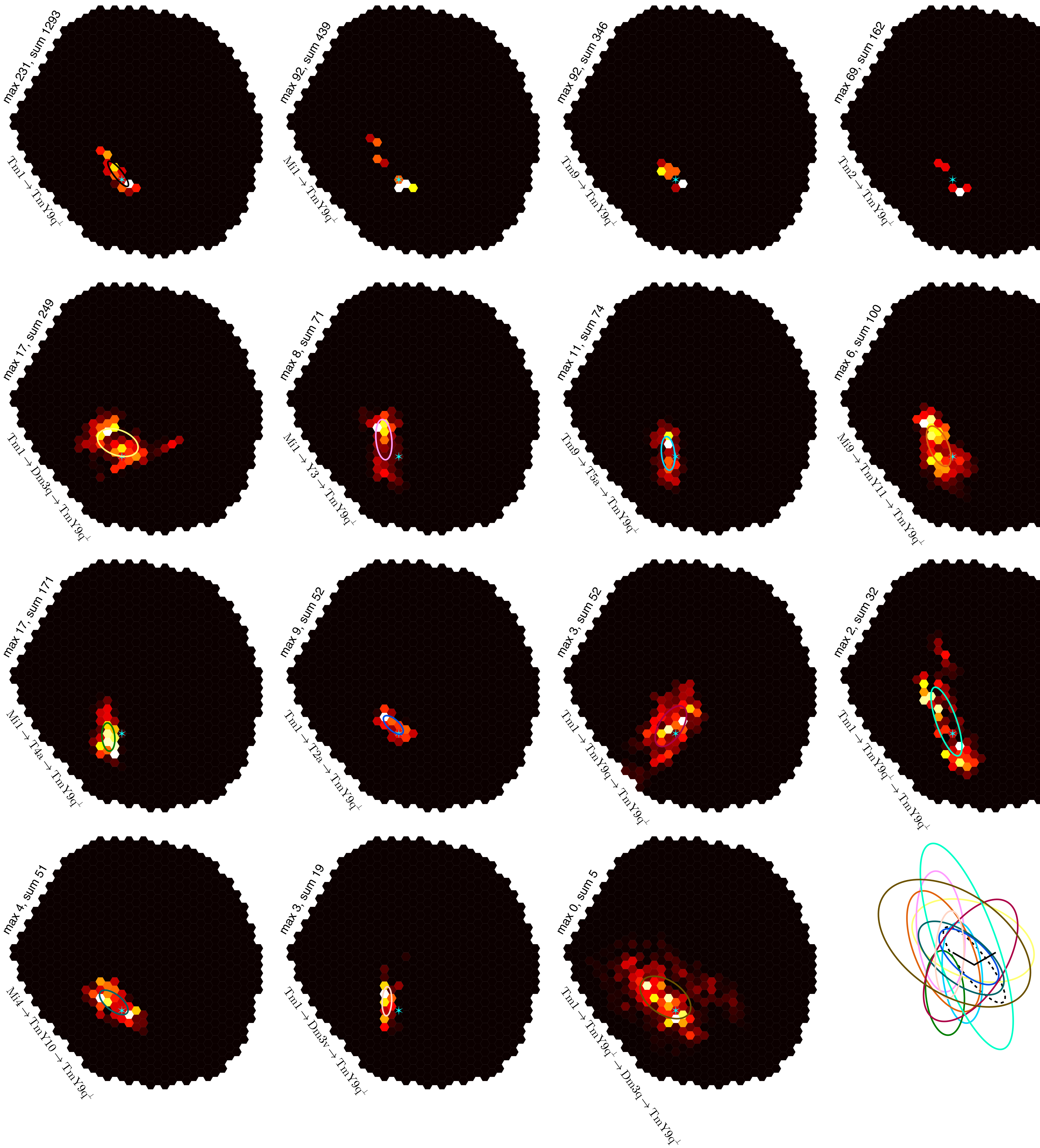
max 231, sum 1293
Tm1 → TmY9q⊥
max 92, sum 439
Mi1 → TmY9q⊥
max 92, sum 346
Tm9 → TmY9q⊥
max 69, sum 162
Tm2 → TmY9q⊥
max 17, sum 249
Tm1 → Dm3q → TmY9q⊥
max 8, sum 71
Mi1 → Y3 → TmY9q⊥
max 11, sum 74
Tm9 → T5a → TmY9q⊥
max 6, sum 100
Mi9 → TmY11 → TmY9q⊥
max 17, sum 171
Mi1 → T4a → TmY9q⊥
max 9, sum 52
Tm1 → T2a → TmY9q⊥
max 3, sum 52
Tm1 → TmY9q → TmY9q⊥
max 2, sum 32
Tm1 → TmY9q⊥ → TmY9q⊥
max 4, sum 51
Mi4 → TmY10 → TmY9q⊥
max 3, sum 19
Tm1 → Dm3v → TmY9q⊥
max 0, sum 5
Tm1 → TmY9q⊥ → Dm3q → TmY9q⊥
